# A spontaneous reduction in tumor size of a thymic carcinoma: a case report

**DOI:** 10.1186/s40792-022-01510-w

**Published:** 2022-08-12

**Authors:** Shoei Kuroki, Takanori Ayabe, Hiroyuki Tanaka, Hiroshi Nakada, Ryo Maeda

**Affiliations:** 1 Department of Thoracic and Breast Surgery, Faculty of Medicine, University of Miyazaki, Kihara 5200, Kiyotake, Miyazaki, 889-1692 Japan; 2 Department of Pathology, University of Miyazaki, Miyazaki, Japan; 3 Department of Radiology, University of Miyazaki, Miyazaki, Japan

**Keywords:** Spontaneous regression, Surgery, Thymic carcinoma

## Abstract

**Background:**

Spontaneous regression of thymic carcinoma is extremely rare. We report a case of a resected thymic carcinoma with preoperative spontaneous regression in a 67-year-old woman.

**Case presentation:**

The patient presented with low-grade fever and anterior chest pain. Chest computed tomography (CT) showed a 55 × 43 mm exophytic heterogeneously enhancing mass showing some areas of necrosis. Chest CT done one day preoperatively revealed that the tumor had rapidly shrunk for one month. Surgical resection was performed to obtain a definitive diagnosis and achieve complete resection, yielding a postoperative diagnosis of thymic carcinoma. The patient survived without signs of recurrence for 12 months postoperatively.

**Conclusions:**

Mediastinal tumors with necrosis demonstrating spontaneous regression should include thymic carcinomas in the differential diagnosis.

## Background

Spontaneous tumor regression is defined as the partial or complete disappearance of a malignant tumor in the absence of treatment [1]. Spontaneous regression is a rare phenomenon, with an incidence of < 0.001 in patients with cancer [2].

Thymic carcinoma is a rare malignant disease, which is typically aggressive, with a 5-year survival rate of only 28–67% [3, 4]. Surgical excision is the gold standard treatment for resectable thymic carcinoma [3, 4]. Herein, we report an extremely rare case of a resected thymic carcinoma that demonstrated preoperative spontaneous regression, and we discuss the mechanism for this rare phenomenon.

## Case presentation

**Fig. 1:**
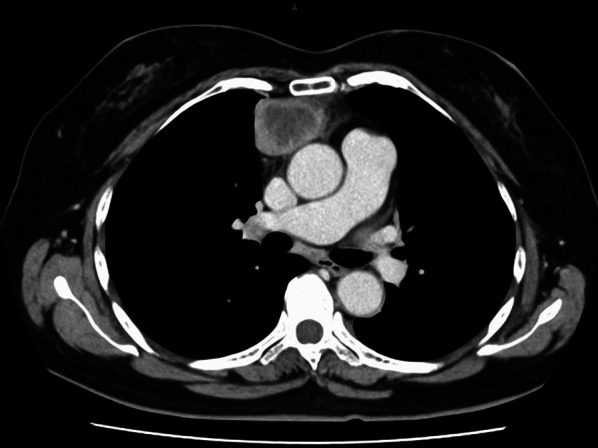
Chest computed tomography scan revealing a 55 × 43-mm exophytic heterogeneously enhancing mass exhibiting some areas of necrosis

**Fig. 2:**
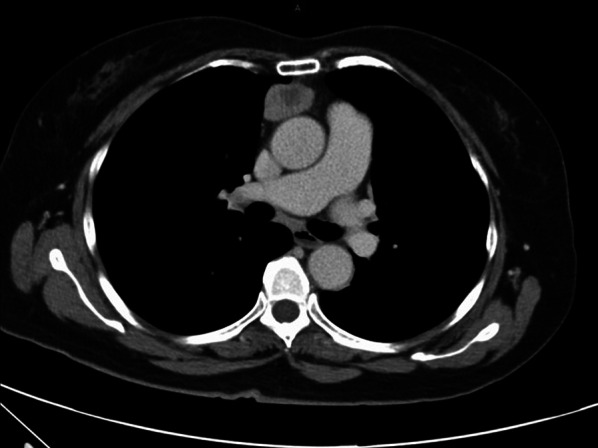
Chest computed tomography scan just before the operation revealing that the mass had regressed in size in the past month

A 67-year-old woman with no past medical history had a chief complaint of anterior chest pain and low-grade fever for 1 week. Chest computed tomography (CT) showed a 55 × 43 mm exophytic heterogeneously enhancing mass with some areas of necrosis (Fig. 1). The tumor markers, carcinoembryonic antigen, cytokeratin fragment 21, progastrin-releasing peptide, α-fetoprotein, and human chorionic gonadotropin, were within the normal range. Because a malignant tumor was suspected and complete resection of the tumor was considered possible, surgery was planned to obtain a definitive diagnosis and achieve complete tumor resection. However, chest CT just one day before the operation revealed that the tumor had rapidly shrunk in just one month (Fig. 2). Her symptoms of fever and chest pain were also alleviated at this time.

**Fig. 3:**
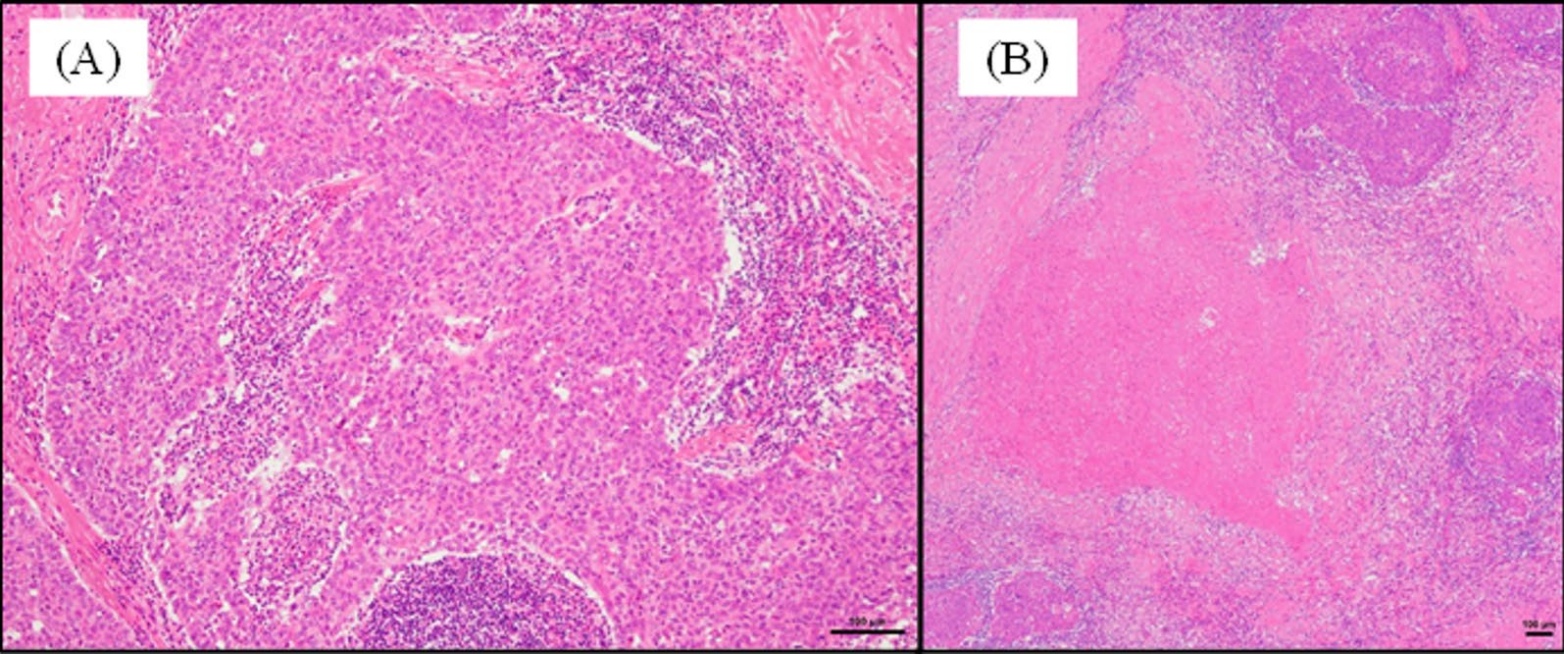
**A** Histopathology revealing squamous cell carcinoma of the thymus (**A**) (hematoxylin and eosin stain) and a large necrotic area in the center of the tumor (**B**) (hematoxylin and eosin stain)

Total thymectomy was performed via video-assisted thoracic surgery. Partial resection of the pericardium was performed because of tumor adhesion. The postoperative histological examination revealed a squamous cell carcinoma of the thymus with minimal invasion into the surrounding thymus and mediastinal fat tissue (stage II in the Masaoka classification) (Fig. 3A). The tumor included large necrotic areas in the center of the tumor (Fig. 3B). Occlusions of the vessels were not found histopathologically. No tumor cells were at the margins of the resected specimen. The postoperative recovery was uneventful. She survived without any signs of recurrence for 12 months after surgery.

## Discussion

Spontaneous tumor regression was first reported by Cole and Everson in 1956 [5] and defined as partial or complete involution of a malignant tumor in the absence of the application of a specific therapy [1]. Spontaneous tumor regression is a rare phenomenon, with an incidence rate of 1 out of every 60,000–100,000 patients with cancer, most commonly seen in malignant melanoma, neuroblastoma, and cancer of the kidney [2].

Thymic carcinoma is a rare carcinoma of the thymus arising in the thymic epithelium, with an incidence of ~ 0.02 per 100,000 person-years [6, 7], and represent > 1% of thymic malignancies [8]. Spontaneous tumor regression is reportedly more frequent in certain malignancies, such as renal cell carcinoma, neuroblastoma, and malignant melanoma [2], whereas it is extremely rare in thymic carcinoma, with only 1 reported case in the English literature [9].

Although it is difficult to determine the scientific and definitive mechanism of the spontaneous regression of cancer, immune mechanisms are considered to play a central role [10, 11] in the observed regression of renal cell carcinoma and malignant melanoma. Other possible mechanisms, which affect spontaneous regression, are as follows: tumor necrosis [12], growth factor, cytokine changes [13], apoptosis [14], psychological factors [15], genetic and epigenetic factors [16], and induction of benign differentiation [17]. In this study, preoperative CT revealed an exophytic heterogeneously enhancing mass exhibiting some areas of necrosis. Some common features of thymic carcinomas include large and highly aggressive anterior mediastinal mass with rapid progression and areas of necrosis [18]. In our case, the resected specimen mostly comprised necrotic components. The phenomenon of spontaneous regression may be related to rapid enlargement because of its highly aggressive characteristics, which could have caused a disorder of the vascular supply, thereby leading to necrosis. Furthermore, this might cause inflammation around the tumor, thereby causing the chest pain.

Surgical excision is the gold standard treatment for resectable thymic carcinoma [3, 4]. Clinicians should recognize the possibility of spontaneous regression among patients with thymic carcinomas; surgical resection is needed even if an anterior mediastinal tumor regresses spontaneously.

## Conclusions

We report a patient with a thymic carcinoma that spontaneously regressed. Mediastinal tumors with necrosis that demonstrate spontaneous regression should include thymic carcinoma in the differential diagnosis, and surgical resection is needed even if the tumor regresses spontaneously.

## Data Availability

Not applicable.

## Abbreviation

CT: Computed tomography
